# Process Improvement Approaches for Increasing the Response of Emergency Departments against the COVID-19 Pandemic: A Systematic Review

**DOI:** 10.3390/ijerph18168814

**Published:** 2021-08-20

**Authors:** Miguel Angel Ortíz-Barrios, Dayana Milena Coba-Blanco, Juan-José Alfaro-Saíz, Daniela Stand-González

**Affiliations:** 1 Department of Productivity and Innovation, Universidad de la Costa CUC, Barranquilla 081001, Colombia; dcoba3@cuc.edu.co (D.M.C.-B.); dstand1@cuc.edu.co (D.S.-G.); 2 Research Centre on Production Management and Engineering, Universitat Politècnica de València, 46022 Valencia, Spain; jalfaro@omp.upv.es

**Keywords:** healthcare, emergency department, COVID-19, process improvement, systematic review

## Abstract

The COVID-19 pandemic has strongly affected the dynamics of Emergency Departments (EDs) worldwide and has accentuated the need for tackling different operational inefficiencies that decrease the quality of care provided to infected patients. The EDs continue to struggle against this outbreak by implementing strategies maximizing their performance within an uncertain healthcare environment. The efforts, however, have remained insufficient in view of the growing number of admissions and increased severity of the coronavirus disease. Therefore, the primary aim of this paper is to review the literature on process improvement interventions focused on increasing the ED response to the current COVID-19 outbreak to delineate future research lines based on the gaps detected in the practical scenario. Therefore, we applied the Preferred Reporting Items for Systematic Reviews and Meta-Analyses (PRISMA) guidelines to perform a review containing the research papers published between December 2019 and April 2021 using ISI Web of Science, Scopus, PubMed, IEEE, Google Scholar, and Science Direct databases. The articles were further classified taking into account the research domain, primary aim, journal, and publication year. A total of 65 papers disseminated in 51 journals were concluded to satisfy the inclusion criteria. Our review found that most applications have been directed towards predicting the health outcomes in COVID-19 patients through machine learning and data analytics techniques. In the overarching pandemic, healthcare decision makers are strongly recommended to integrate artificial intelligence techniques with approaches from the operations research (OR) and quality management domains to upgrade the ED performance under social-economic restrictions.

## 1. Introduction

The SARS-CoV−2 (COVID-19) disease first appeared on 30 December 2019, in Wuhan, China [1], and continues to affect the global population in the near future. The clinical signs of COVID-19 range from unapparent non-symptomatic infection to severe pneumonia and death [2]. From a healthcare perspective, the swift and unpredicted spread of COVID-19 throughout the world in nearly four months in 2020 has significantly influenced the emergency departments (EDs) of the affected countries in view of the large, irregular, and overwhelming number of infected people to attend. The COVID-19 impact is even more severe in regions with infrastructure restrictions and low preparedness in their EDs to address disasters, which has contributed to greater infection and mortality rates in contrast with more developed countries [3]. As of 20 May 2021, it has caused approximately 164.5 million cases and 3.4 million deaths in the world [4] without considering the huge drain on the financial resources of emergency care systems.

The here-described context has forced many EDs to operate at full capacity or near full capacity. In pandemic conditions where demand peaks are expected, EDs may find it arduous to perform their functional activities, and installed capacity may no longer satisfy this demand [5]. An evidence of this is the aggravation of waiting times and overcrowding experienced by the patients as well as the high left-without-being-seen rates (LWBS) observed in the emergency care wards [6]. There is also a worrisome upward trend to receive COVID-19 patients in a more critical health condition which, added to the increased demand, poses a significant workload on EDs around the world. Being aware of this situation, the decision makers are then advised to rapidly deploy robust emergency care configurations to lessen the effects of the pandemic [7], thereby alleviating the burden faced by the stakeholders (i.e., health professionals, COVID-19 patients, healthcare authorities) involved in this service as the pandemic evolves. The accelerating nature of the COVID-19 virus demands that flexible EDs rearrange their systems to allocate scarce resources to save the highest number of lives [8,9].

Despite the aforementioned scenario, most of the efforts against COVID-19 carried out by governments, industry, and academia tend to focus only on monitoring the health conditions of COVID-19 patients using connected health technologies [10]. Likewise, little work has been carried out on the creation and deployment of strategies dealing with the operational inefficiencies detected along the patient journey. In this regard, Hundal et al. [11] proposed the use of lean six sigma to tackle the overriding patient safety domain while supporting the supply chain resilience behind the emergency care operations. On the other hand, Gupta et al. [12] identified how different operations management techniques can provide a solution for outstripping a range of ED challenges derived from the current pandemic scenario. The reported literature then evidences no reviews presenting methodological approaches that can be used by ED decision makers to deal with the fast and unexpected COVID-19 global spread and its widespread disruption on ED functioning. This paper therefore bridges these gaps in evidence by a systematic review directed towards establishing the (i) most popular ED operational aims targeted during the COVID-19 outbreak, (ii) the process-improvement techniques that have been frequently used by practitioners to increase the ED response against the pandemic, (iii) trends in related publication as COVID-19 evolves, and (iv) the journals most contributing to the groundwork of applications aiming at improving the ED dynamics when treating COVID-19 patients. Our article then provides a solid framework for examining the progress of this research field, designing cost-effective interventions upgrading the ED performance against the COVID-19 outbreak, pointing out gaps in the practical scenario, and devising opportunities for future research. In summary, this review was performed to address the following Population-Intervention-Comparators-Outcomes (PICO) question: What process improvement approaches from the industrial engineering domain have EDs implemented to increase their operational response in managing COVID-19 patients?

The rest of the paper is organized as follows. Section 2 describes the framework followed to perform the systematic review whereas Section 3 presents the results. Finally, conclusions and future research lines are outlined in Section 4.

## 2. Methods

This review was performed and reported considering the Preferred Reporting Items for Systematic Reviews and Meta-analyses (PRISMA) structure:

### 2.1. Search Strategy and Information Sources

The PICO declaration was employed to establish the eligibility criteria underpinning the review in order to pinpoint the population, intervention, comparators, and outcomes [13]. The ISI Web of Science, Scopus, PubMed, IEEE, Google Scholar, and Science Direct databases were chosen in view of the wide coverage of Health Sciences, Industrial Engineering, Software Engineering, and Management literature. This review was not recorded in any database.

**Figure 1:**
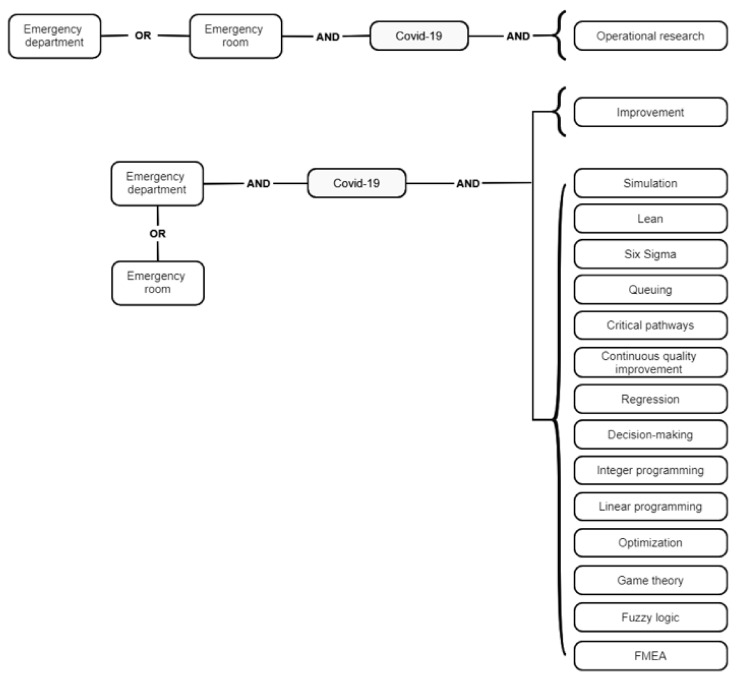
Search algorithms used in the literature review.

We constrained our comprehensive literature examination to full-text case articles from December 2019 (the date on which the pandemic was initiated) and April 2021 evidencing process improvement methodologies implemented by decision makers, policymakers, ED administrators, practitioners, and researchers to upgrade the response of EDs when managing COVID-19 patients. The papers had to be written in English and provide data grounding the outcomes achieved during the interventions. Research papers depicting conceptual frameworks without application in the practical scenario were not considered in this review. Additionally, conference articles, doctoral theses, textbooks, master’s dissertations, and review papers were discarded. The search strategy included the codes (Figure 1) considering the most prominent process improvement techniques identified in Ortíz-Barrios and Alfaro-Saíz [6] as well as keywords related to the topic of review. The techniques included here can serve as a methodological framework supporting interventions undertaken by healthcare administrators, ED managers, health authorities, and practitioners involved in the strategic and tactic decision levels of the emergency care system. These terms are also familiar for these stakeholders as evidenced in Elamir [14] (lean), Rotteau et al. [15] (continuous quality improvement), Cheng et al. [16] (regression), Ashour and Kremer [17] (simulation), Feng et al. [18] (optimization), Bellew et al. [19] (critical pathways), Youseffi and Ferreira [20] (decision making), Nezamoddini and Khasawneh [21] (integer programming), Bish et al. [22] (queuing), Blick [23] (2013) (six sigma), Azadeh et al. [24] (fuzzy logic), Acuna et al. [25] (game theory), Sorrentino [26], and Saghafian et al. [27] (operational research) where real applications have been fully reported before the pandemic era with the participation of different healthcare workers, especially those from the managerial dependencies. Additionally, we performed a manual search taking into account the references related to the reviewed manuscripts. The use of the “improvement” keyword and the ample number of techniques from the industrial engineering area considered in this review also ensures a significant literature coverage granting the identification of manuscripts with great contribution to the evidence base.

### 2.2. Selection Process and Data Extraction

Specifically, the selection process and data extraction were performed by three independent reviewers (M. O., D. C., and D. S.) who profoundly examined the papers in their full length considering the criteria for exclusion and inclusion outlined in Section 2.2.1. Opposite positions among reviewers regarding the inclusion of a manuscript were addressed via discussion and consensus. The manuscripts were later enlisted in a data extraction template containing the paper title, authors, publication date, journal, process-improvement approaches used, approach nature, and primary aims. The articles were then classified into four strategic categories (quality management, operational research, machine learning and data analysis, and design and implementation of protocols) representing the research field that was employed by authors for increasing the response of EDs against COVID-19. In this regard, some papers evidenced the use of approaches from two domains and were therefore included in both classifications. The process improvement techniques, either single or hybrid, used in these applications were also specified. Moreover, the selected papers were further categorized considering the publication time to identify trends and patterns in the reported related literature.

#### 2.2.1. Criteria for Including Studies in this Review

##### Types of Studies

We particularly considered manuscripts evidencing the implementation of an industrial engineering approach for increasing the operational response of a real emergency department during the current COVID-19 outbreak. The studies had to report high-quality data supporting the interventions as well as the outcomes achieved after implementation. Controlled before-and-after studies (CBAs) and randomized controlled trials (RCTs) satisfying the quality criteria outlined by the Cochrane Effective Practice and Organization of Care Group (EPOC) [28] were principally taken into consideration. Manuscripts only limited to descriptive statistics and/or no application in the wild were discarded.

##### Types of Participants

We included EDs of any healthcare level caring for suspected/confirmed COVID-19 patients.

##### Types of Interventions

We took into account applications from the industrial engineering domain aimed at ramping up the operational response of EDs against COVID-19. The improvement interventions were classified based upon the following research domains:

Operational research (OR) is a discipline employing analytical and soft techniques capable of supporting decision making within the healthcare domain and specifically unraveling the dynamics of COVID-19 and its effects on the emergency care provision [29]. Additionally, OR can offer quantitative evidence to deploy scale-up interventions along the ED patient journey while administering interactions among health centers [30,31].

Quality management (QM) is a broad theme encompassing a set of approaches focused on the continuous improvement of emergency care towards maximizing customer satisfaction. QM can be only derived from planned management action which entails a certain degree of quality culture within the institutions leading the emergency care services [32].

Machine learning and data analytics (MLDA) is based on learning models integrated by inputs categorized as predictor measures, and outputs, representing the optimal solution [33]. On the other hand, the big data analysis tool contains a set of complex data nested to the application of artificial intelligence algorithms and automated learning with high potential in ED healthcare. This is of fundamental support for health personnel in operations planning and implementation, decision making, disease detection, and other areas; thereby contributing to the increase in the quality of care provided in EDs and the intricate reduction of costs [34].

Protocol design and implementation (PDI) is related to the procedures carried out in the EDs with a particular view on their design and implementation. ED protocols are called to strengthen the response of the emergency care units considering the practices undertaken by the health personnel [35] and the communications flows within the ED teams [36].

We also deemed a standard practice comparator (e.g., no intervention/routine practice or another approach) or any type of ongoing strategy targeting improved ED operational response during the pandemic.

##### Outcome Measures

We included performance indicators that are commonly used in different ED stages and/or patient evolution pathways. Some of them are:

✓ Mean length of stay (LOS)
✓ Left-without-being-seen rate (LWBS)
✓ Average flow time
✓ Median time to ED revisit
✓ Median waiting time for consultation

Additionally, there are measures linked to the particular COVID-19 pandemic (i.e., % of available ventilators, number of patients with unfavorable outcomes, predicted number of COVID-19 patients, median time to COVID-19 results) and new safety outcomes (i.e., COVID-19 infection rate within the ED wards). The costs derived from the emergency care attention during the COVID-19 pandemic were not determined in this review.

### 2.3. Risk of Bias Assessment

Three independent reviewers (M. O., D. C., and D. S.) appraised the risk of bias in each paper according to Balini et al. [37] and EPOC [28]. A total of 8 evaluation factors were considered: (i) random sequence generation, (ii) allocation concealment, (iii) baseline outcome measurements similar, (iv) baseline characteristics similar, (v) incomplete outcome data, (vi) knowledge of the allocated interventions adequately prevented during the study, (vii) selective outcome reporting, and (viii) other risks of bias. A grade was assigned to each manuscript taking into account the following scoring system:

✓ Low risk: all the factors were graded as “low risk”
✓ Moderate risk: one or two factors were qualified as “unclear risk” or “high risk”
✓ High risk: more than two factors were graded as “unclear risk” or “high risk”

### 2.4. Effect Measures

For CBAs and RCTs, we provided different comparison measures representing the extent at which the improvement was achieved. The set of indicators include:

✓ Mean length of stay (LOS) (percentage difference; absolute change with 95% confidence interval, inter-quartile range—IQR)
✓ Left-without-being-seen rate (LWBS) (average rate difference)
✓ Average flow time (absolute difference)
✓ Median time to ED revisit (median difference)
✓ Median waiting time for consultation (percentage change)
✓ % of available ventilators (percentage difference)
✓ Number of patients with unfavorable outcomes
✓ Predicted number of COVID-19 patients (percentage difference, absolute change with 95% confidence interval, *p*-value)
✓ Median time to COVID-19 results (IQR, absolute change with 95% confidence interval)
✓ COVID-19 infection rate within the ED wards (percentage difference)

### 2.5. Dealing with Missing Data

In this case, all the selected studies completely reported the data of interest; it was therefore unnecessary to contact authors for additional information.

### 2.6. Heterogeneity Evaluation

We descriptively outlined heterogeneity of selected studies via appraising differences in terms of primary aims, process-improvement approaches used in the interventions, contributing research domain, and outcomes.

### 2.7. Data Synthesis, Summary Tables, and Confidence Assessment

We qualitatively depicted the results of the selected studies. In this case, meta-analysis was not possible considering the significant statistical heterogeneity and variability of the interventions (methodological diversity) identified in the selected studies. Furthermore, the outcomes presented in the reported related literature are too diverse; therefore pooling and analyzing the combined data is not appropriate [38]. We did not carry out any sensitivity analysis. Instead, evidence tables are provided in Section 3.1 and Section 3.2. for authors to review data and principal outcomes of the manuscripts included in the review. In addition, summary tables indicating the techniques employed in each study were displayed complemented by graphs depicting the most used approaches, the most popular primary aims, and the evolution of the research body against time. On the other hand, we assessed the quality of evidence for the outcomes provided in each manuscript by using the GRADE method [37]. The evaluation scale is as follows: “Very low”, “Low”, “Moderate”, and “High”.

## 3. Results

**Figure 2:**
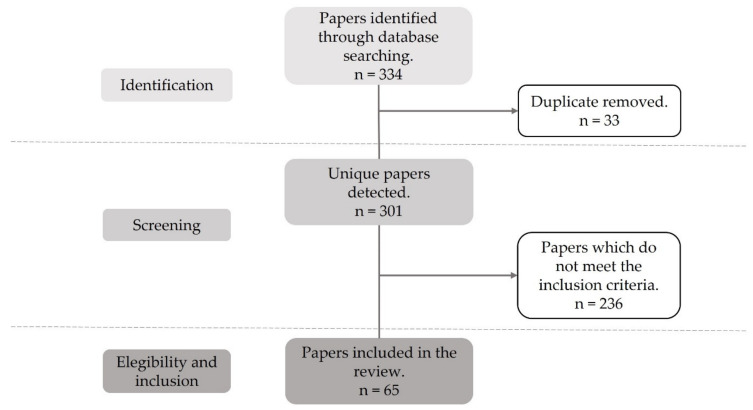
PRISMA flow diagram.

In this section, we present the results of the search and selection process based on the search codes shown in Figure 1. After removing the duplicates (*n* = 33), the resulting papers (*n* = 301) were exhaustively screened to select the relevant papers meeting the above-mentioned inclusion criteria. As a result, 65 papers were found to satisfy the inclusion requirements. Most of the discarded manuscripts were merely descriptive, did not present data supporting the intervention outcomes or evidenced approaches out of the industrial-engineering domain. The resulting PRISMA scheme can be observed in Figure 2.

### 3.1. Study Characteristics, Quality of the Evidence, and Risk of Bias

Table 1 details the characteristics of the papers that were finally included in the review. In particular, the outcomes pursued in each study and the sample supporting the intervention are presented. Sample values without units are assumed to be the number of patients enrolled in the project. In some cases, the sample size was too small which may limit the applicability of the related proposed methods in the practical scenario. On the other hand, the outcomes revealed in the body of evidence were noted to be ample and diverse which denotes the multidimensional nature of emergency care operations and the evolving dynamics of the COVID-19 outbreak. In fact, new measures have been created to evaluate the effectiveness of the proposed process improvement approaches. Likewise, the papers were highly heterogeneous based upon the different types of interventions and clinical aspects that became glaring from the literature and thus making it unfeasible to undergo a meta-analysis. On a different tack, most of the comparators (*n* = 53 articles; 81.53%) were found to be “Routine practice” (no intervention) which is expected given the recentness of the pandemic scenario. A challenge, however, is posed regarding the implementation of comparative studies delineating an alternative avenue through the context in which ED operations are now being performed. The key findings and conclusions are also outlined in this table for supporting practitioners, researchers, and ED administrators in plotting actions directed towards better operational response against the COVID-19 outbreak. The majority of interventions evidenced in the literature are data-grounded which also facilitates their adoption and replication in the wild; likewise, the upheaval required for more aggressive and agile methodologies reducing the waiting times in consideration of a rapidly evolving disease was glaring.

On a different note, the GRADE approach was utilized to appraise the quality of the related evidence detected through this review. In this case, most of the studies ranged from “low” (*n* = 23 studies; 35.38%) to “moderate” (25 studies; 38.46%). Therefore, researchers leading future related studies should adhere to the quality criteria described by EPOC (2021) [28], stressing upon, for instance, the use of sample sizes with high statistical significance so that reliable outcomes can be properly derived. Indeed, very few studies demonstrated how the sample size supporting the conclusions was estimated.

**Figure 3:**
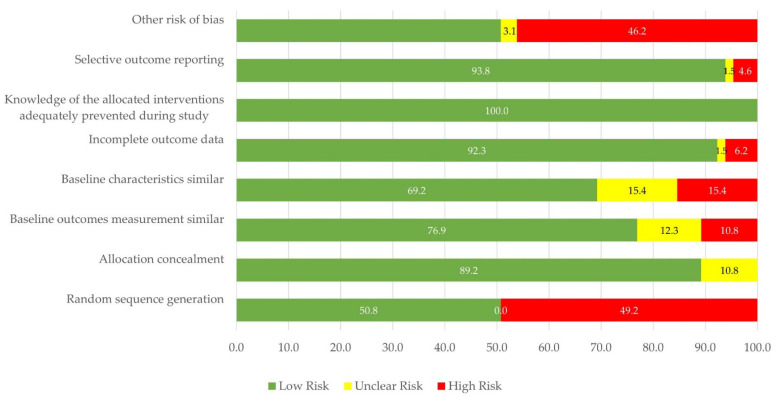
Risk of bias graph—an analysis of the 8 evaluation criteria.

Arguably, evaluating the risk of bias is another important aspect to be addressed in this review. In response to this important task, we used the approach illustrated in Section 2.3 whose results were included in the first column of Table 1. Specifically, one asterisk (*) denotes “low risk”, two asterisks (**) represents “unclear risk”, while three asterisks (***) symbolizes “high risk”. In this review, most of the studies (*n* = 38 studies; 56.46%) were categorized as “unclear risk” whereas 21.53% (*n* = 14 studies) were found to have “low risk). A deeper view of this evaluation can be noted in Figure 3 where results on the eight domains are widely reported. Based on the graph, it can be seen that more than 50% of the studies were found to have a low risk of bias in all the domains. Special attention should be paid to the “other risk of bias” whose “high risk” participation corresponds to 46.2% of the total evidence inasmuch as limitations regarding the use of small sample sizes, no proper handling of missing data, and no inclusion of critical variables were frequently reported in the “limitations” sections of these papers. An important portion of papers (49.2%) was also informed to have a “high risk” of bias given the no employment of random sequences in their study designs. These methodological aspects need to be tackled by future practitioners and decision makers to ramp up the validity and effectiveness of the interventions.

### 3.2. Classification Schemes

We then categorized the selected papers based upon the classification schemes considered in the review: (i) contributing research domain, (ii) primary aim, (iii) publication period, and (iv) contributing journal. The findings will guide researchers, ED administrators, health authorities, and practitioners in the design of strategies increasing the response of emergency care processes against the inherent operational demands of the COVID-19 outbreak as well as the definition of future research lines.

#### 3.2.1. Classification Based on the Contributing Research Domain

The complexity and multi-causality nature of EDs and the devastating spread of COVID-19 demand for solutions integrating different research themes from the industrial engineering domain. Thereby, it will be possible to support the decision-making process within these units and hence increase their response against the current pandemic. In this review, the four research domains outlined in Section 2.2.1. were considered.

##### Techniques from the Operational Research Domain

Table 2 shows the classification of research papers from the operational research domain evidencing increased responses in the emergency department. COVID-19, as a threat to worldwide public health, has generated an overflow of emergency units, which can be effectively addressed through OR methods. On the other hand, Table 2 depicts the techniques used in each study while specifying whether a single or hybrid (combination of two or more techniques) approach was employed. As a result, a total of 16 articles (24.61%) were found to apply OR techniques. From these, half (*n* = 8) evidenced the use of approaches combining two techniques with no trend in a particular integration.

The techniques used in studies with single approaches are varied and respond to the complex nature of emergency care processes and their interactions. An example can be found in Tang et al. [93] where the authors employed stochastic simulation to appraise the performance of five different staffing options in terms of LOS and LWBS rates. The results revealed that incorporating an extra ED provider between standard ED rooms would lead to the most significant decrease in LOS for both admitted and discharged patients. Similarly, Peng et al. [85] built a simulation model of a local ED to improve the patient flow efficiency. Bottlenecks in the emergency care process were detected and alternative strategies were devised to reduce the patient waiting time and LOS. The proposed method can be further replicated from a sectorial perspective to improve the operational efficiency of emergency care systems during the current COVID-19 pandemic. In turn, Moss et al. [80] simulated clinical presentations and patient flows through the Australian health care system, including expansion of available acute care capacity and alternative clinical assessment pathways. Other interesting related studies are illustrated in Nepomuceno et al. [81], Mehrotra et al. [77], AbdelAziz et al. [40], Aggarwal et al. [41], and Araz et al. [45].

On a different tack, different interventions employed OR techniques in a paired way. For instance, Zeinalnezhad et al. [98] implemented colored petri nets and discrete event simulation to prepare hospitals for a virus outbreak. The initial simulation of current cardiac clinic processes first identified the ED bottlenecks. Their analysis confirmed that the current workflow was not optimal for COVID-19 patients; three optimization scenarios were therefore proposed to reduce the waiting time problem. In a different study, Haddad et al. [67] crafted a decision-making approach for the creation of local emergency response manufacturing networks reducing shortages of medical supplies in times of COVID-19 crisis. In this work, interrelated simulation and stochastic models are applied to optimize the ventilator allocation in USA emergency departments considering uncertainty. In another study, Abadi et al. [39] presented a novel integration between the hybrid swarm salp and genetic algorithm (HSSAGA) to define nurse scheduling and designation during the COVID-19 period. Their findings revealed that this approach outperformed other techniques frequently employed in the literature for the nurse scheduling problem. The proposed framework provides ED managers with an intelligent automated framework capable of eliminating exposed shifts while mitigating low nursing staff commitment and stress. Other highlighted interventions are reported in Garbey et al. [62], Albahri et al. [42], De Nardo et al. [58], Parker et al. [84], and Zhang & Cheng [100].

##### Techniques from the Quality Management Domain

Quality management contributions have become a cornerstone for the design and deployment of operational solutions in different healthcare services due to their capability of reducing flow times and cost overruns while satisfying patients’ expectations [37]. Table 3 shows the compiled efforts of distinct authors who have used methods from this domain to upgrade the performance of EDs during the COVID-19 period. Several studies have presented the implementation of these approaches in the real context of ED with particular attention to optimizing processes, reducing failures, and efficiently managing resources. The evidence base (*n* = 6 articles; 9.23%), however, is still scant and less popular compared with those from OR and MLDA. 66.6% (*n* = 4 articles) of the total related body of knowledge utilized a single technique for dealing with different ED performance challenges during the current pandemic.

An interesting QM-based work is reported in Balmaks et al. [48] who analyzed the performance gaps and failures generated in their system through a multicenter and cross-sectional study based on the simulation of COVID-19 demands and their effects upon the normal ED operation. On a different tack, Teklewold et al. [94] implemented the failure mode and effect analysis (FMEA) tool to improve the pandemic management in EDs. In this document, impacts on the identification of faults in a showcased hospital ED, as well as possible advanced solutions propelling the reduction of COVID-19 transmission within the health personnel and patients are devised. Additionally, we identified a research authored by Chen et al. [53] using lean manufacturing to upgrade the adjusted workflow and value flow of an ED. Similarly, Casiraghi et al. [52] used FMEA for early evaluation and rapid risk prediction, which contributed to more successful diagnosis and consequently increased the likelihood of complete recovery.

In light of Table 3 results, it is evident that only two articles reported the combination of QM methods with other approaches for tackling poor ED performance against the operational COVID-19 implications. On one hand, Retzalff [21] applied lean manufacturing and protocol design for supporting the creation of critical units and the strategies implemented for dynamic protocol change within the hospital; thereby facilitating visibility and training for health workers. For O’Reilly et al. [83], the improvement of care and emergency processes lie in the restructuring of system design, resource allocation, and clinical management during the pandemic, based on the premise of machine learning and continuous quality improvement techniques for data analysis and decision making.

##### Techniques from the Machine Learning and Data Analytics Domain

The application of MLDA techniques has been widespread to solve a wide range of inefficiencies in healthcare services [43]. Prevention is always better than correction and the use of predictive models can foster the implementation of this policy within the emergency wards. The increasing trend in the use of these techniques is supported by the availability of large complex health records that facilitate the design of targeted interventions based on data patterns. Accordingly, we have enlisted the contributions using MLDA methods for upgrading the reaction of EDs against this disastrous event (Table 4). We seek to provide a groundwork for reducing the knowledge gap that exists regarding the identification of patient COVID-19 risk while ensuring the fastest diagnosis. The evidence indicates that MLDA domain is the most used when dealing with the ED improvement objective (*n* = 41 articles; 63.07%).

In particular, 53.65% (*n* = 22 articles) employed a single MLDA technique to address the operational problems of EDs during the current COVID-19 pandemic. For instance, Chopra et al. [54] studied the incidence of clinical and demographic risk factors for COVID-19 patients visiting emergency rooms, using multivariate logistic regression models. The results achieved in this work highlighted the importance of using the serum biomarker data to stratify COVID-19 patients into different levels of severity. Another related study was reported by Plante et al. [86] who developed a machine learning model to rule out COVID-19. In a similar vein, Sung et al. [92] confirmed that early and appropriate identification and isolation of patients with suspected COVID-19 is essential to enable timely treatment, optimize resources, protect patients and healthcare workers, and prevent the spread of COVID-19 in healthcare facilities. In this respect, univariate and multivariate logistic regression were used for patient prediction and risk scoring as a complementary tool to help clinicians in triage, quarantine, and testing of suspected COVID-19 patients. Likewise, Nguyen et al. [82] employed a nomogram to identify prognostic factors to predict patient risk and subsequently support the early detection of patients at risk of worsening, improve clinical care, and set out focused therapies. Furthermore, COVID-19 brought about a change in the practices performed in the ED, increasing the workload in such a way that a new distribution is generated; in this regard, Liu et al. [75] proposed an intelligent quarantine station reducing the processing times experienced along with the ED patient journey. Other works presenting solutions from the MLDA domain can be consulted in Kirby et al. [71], Joshi et al. [69], and Van Klaveren et al. [95] who performed a logistic regression model to identify variables associated with discharge appropriateness and optimization of healthcare ED resources during the pandemic. Other useful single-approached proposals can be found in Alfaro-Martinez et al. [43], Freund et al. [61], Brendish et al. [49], Esposito et al. [59], Gordon et al. [66], Levine et al. [74], Carlile et al. [51], and García de Guadiana-Romualdo et al. [63].

On a different note, other interventions considered the need for implementing a hybrid framework for dealing with the COVID-19 problem in the ED context (48.8%; *n* = 21 articles). Indeed 47.6% (*n* = 10 articles) of the hybrid-approached studies were based on two techniques, 33.3% (*n* = 7 articles) employed a mix of three techniques, 9.5% (*n* = 2 articles) used four methods, whereas five and six techniques were used in only one publication each. An example can be observed in Shamout et al. [89] who proposed an automatic risk prediction based on a deep neural network deeming X-ray images. The outcomes are promising in view of assisting the frontline physicians when triaging COVID-19 patients. Furthermore, Balbi et al. [47] used Poisson and logistic regressions with the horizon of assessing inter-rater agreement of initial radiographic findings in COVID-19 patients brought to ED presentation. These results can help to identify patients at risk of death and determine ventilatory support requirements which are even more useful in those ED settings with a high prevalence of the disease. On the other hand, the need for immediate clinical decision making and efficient use of healthcare resources prompted De Moraes et al. [57] to predict the risk of a positive COVID-19 diagnosis by a multiple machine learning approach using only ED admission test results as predictors. Meanwhile, Heldt et al. [68] combined logistic regression, random forest, and gradient-boosting decision tree to underpin early risk evaluation from COVID-19 patients attending the EDs. A novel aspect here is the inclusion of data recently collected during the admission period. A different perspective is noted in Zhang et al. [99] who provided an epidemiological bi-domain integration between the Markov model and logistic regression intending to determine the duration and trend of COVID-19, analyze the general clinical characteristics, and define prevention methods. Other studies combining different MLDA methods are presented in Zhang & Cheng [100], O’Reilly et al. [83], Assaf et al. [32], Chou et al. [55], Van Singer et al. [96], McDonald et al. [76], Möckel et al. [79], Diep et al. [56], Saegerman et al. [88], Romero-Gameros et al. [87], Bolourani et al. [50], Goodacre et al. [65], Feng et al. [46], and Gavelli et al. [64].

##### Techniques Related to Protocol Design and Implementation

Healthcare protocols play a pivotal role in the battle against the current COVID-19 pandemic in view of the need for reducing the virus transmissibility and the inherent mortality risk of both health workers and the community. Well implemented, these guidelines properly endorse the correct administration of factors potentially causing adverse events, an aspect of extreme importance when addressing a pandemic situation. This of course facilitates fruitful teamwork as well as the rapid acquisition of competencies required by the clinicians and administrative staff within the framework of an integrated emergency care response. In this review, the studies using PDI techniques represent 7.69% (*n* = 5 articles) of the total related research (Table 5). Most of the applications (*n* = 4 articles; 80%) addressed the low ED response by a single technique while only the study reported by Retzlaff [35] used two methods for solving this problem.

Most single-approached studies evidenced the use of critical pathways for tackling the unsuitable reaction of EDs against the pandemic. For instance, Suh et al. [91] designed a clinical crisis pathway for risk stratification of COVID-19 patients considering symptomatology, resource allocation, and operational capacity of the emergency system. Furthermore, the study carried out by Sangal et al. [36] denotes the implementation of a new triage system responding to the pandemic demands in a timely manner. This redesign contributed to the optimization of patient flow and ED infrastructure which is highly required in times where physical expansions are not feasible due to the emerging financial constraints. Likewise, Sherren et al. [90] developed a pathway-focused on air failure management during the COVID-19 period with particular attention towards the installed capacity of critical care units. A distinct proposal is presented by Mitchel et al. [85] whose strategies are centered on moving shifts, lessening non-essential personnel, and isolating the COVID-19 patient areas. The primary results revealed the benefits in the matter of bed allocation and reduced overcrowding in the ED corridors. Finally, a hybrid approach combining two domains (QM and PDI) is fostered by Retzlaff [35]. Specifically, this paper compiles the strategies crafted by different USA hospitals to halt the knock-on effects of COVID-19 on the normal ED operation. As this is a rapidly evolving situation, authors recommend using lean manufacturing and healthcare protocols due to their easiness of implementation in the real world in addition to bolstering the internal communication, teamwork, and leadership mainly required in this context.

#### 3.2.2. Classification Based on the Primary Aim

**Figure 4:**
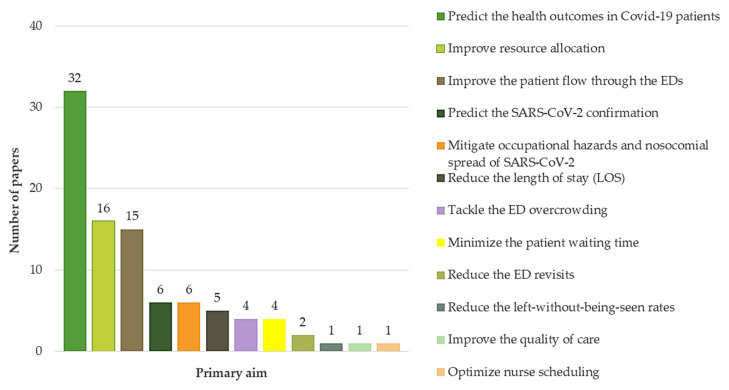
The most popular primary objectives pursued by EDs during the COVID-19 pandemic.

Different specific objectives have been targeted by decision makers when searching for process improvement methods upgrading the performance of EDs against the COVID-19 pandemic. In summary, 12 primary aims were identified from the related reported literature. Figure 4 depicts the most popular primary aims pursued during the implementation of the methodological frameworks (single or hybrid) outlined in Section 3.1. From Figure 4, it can be inferred that approximately half of the studies (*n* = 32 articles; 49.23%) aimed at predicting the health outcomes in COVID-19 patients, whereas 24.61% (*n* = 16 articles) focused on improving resource allocation and improving the patient flow through the EDs (*n* = 14 articles; 21.53%), and were ranked second and third, respectively. On the other hand, very few studies have been published regarding the following aims: (i) reduce the left-without-being-seen rates (*n* = 1 article; 1.53%), (ii) improve the quality of care (*n* = 1 article; 1.53%), (iii) optimize nurse scheduling (*n* = 1 article; 1.53%), (iv) reduce the ED revisits (*n* = 2 articles; 3.07%), (v) minimize the patient waiting time (*n* = 4 articles; 6.15%), (vi) tackle the ED overcrowding (*n* = 4 articles; 6.15%), (vii) reduce the LOS (*n* = 4 articles; 6.15%), (viii) predict the SARS-CoV−2 confirmation (*n* = 6 articles; 9.23%), and (ix) mitigate occupational hazards and nosocomial spread of SARS-CoV−2 (*n* = 6; articles; 9.23%).

**Figure 5:**
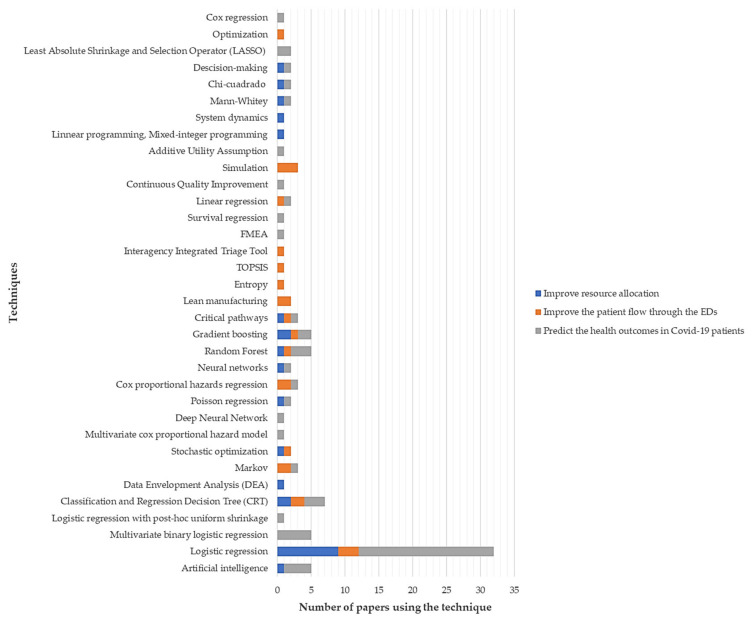
The techniques used to address the most popular objectives pursued by ED decision makers during the pandemic.

Interestingly, logistic regression (Figure 5) has been the most popular technique for predicting the health outcomes in COVID-19 patients (*n* = 20 articles; 62.5%) while variations of this method (multivariate binary logistic regression and logistic regression with post-hoc uniform shrinkage) represent 18.8% (*n* = 6 articles) of the interventions targeting this aim. Similarly, logistic regression has been the most used methodology for improving the resource allocation within the EDs (*n* = 9 articles; 56.25%). On the other hand, logistic regression and simulation (*n* = 3 articles; 21.4%) were the most popular methods when improving the patient flow throughout the ED journey. It is evident that multiple methodological options have been adopted for dealing with the latter objective. This is also noted, albeit to a lesser extent, in the rest of the aims which confirms the complex nature of ED operations, especially in the ongoing pandemic scenario.

#### 3.2.3. Classification Based on the Publication Period and the Contributing Journal

**Figure 6:**
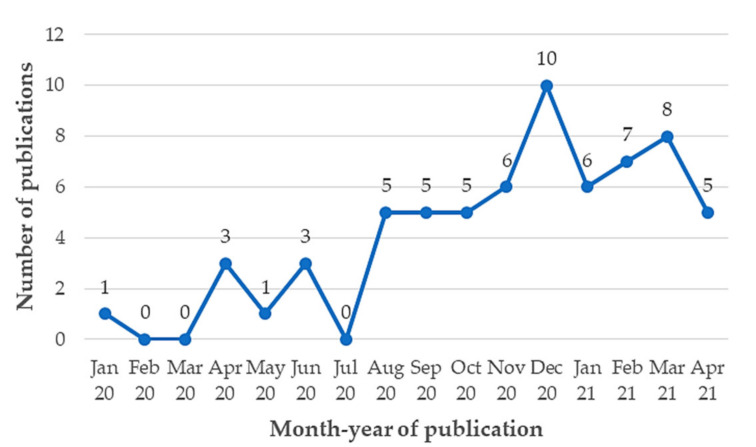
Number of papers evidencing the use of process improvement approaches for ramping up the response of EDs against COVID-19 (Period: January 2020–April 2021).

Figure 6 evidences the evolution of interventions focusing on augmenting the ED performance in response to the pandemic. As expected, the number of papers dealing with this objective was very low at the beginning of this disastrous event (January 2020–July 2020) which evidences the early-stage nature of this research field. In fact, the maximum number of publications was three (April 2020, June 2020). Nevertheless, there is an increasing trend in the number of papers from August 2020 to April 2021 which denotes a growing interest in searching for methodological solutions tackling the above-mentioned challenge. In this period, the publication peaks are observed in December 2020 (*n* = 10 articles) and March 2021 (*n* = 9 articles).

**Figure 7:**
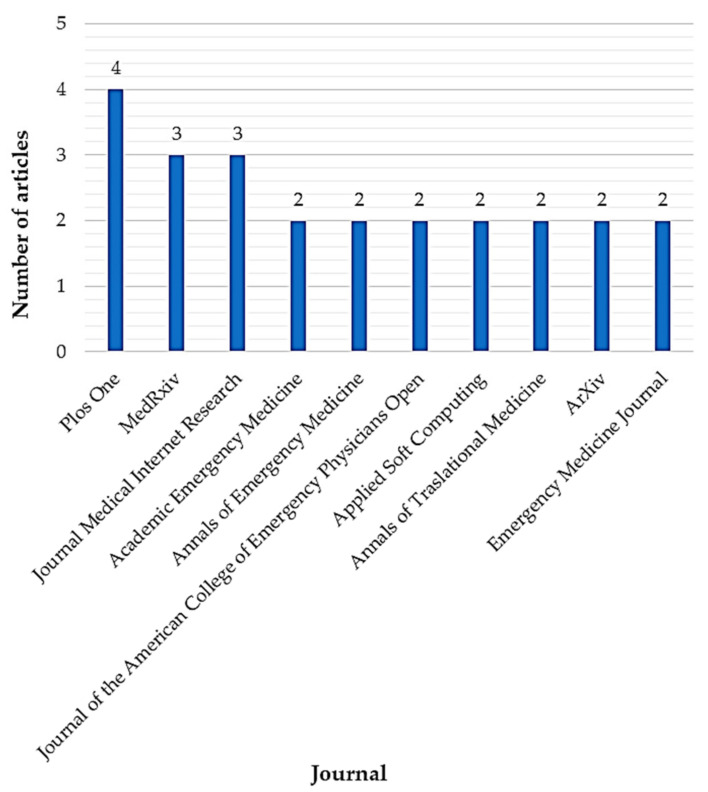
The journals most contributing to the evidence base.

On a different tack, Figure 7 enlists the ten most contributing journals involved in the research field together with the number of selected articles that have published until April 2021. In this case, the evidence base is widespread in 51 journals from different areas of knowledge (i.e., *Health Sciences*, *Industrial Engineering*, *Software Engineering*) which is supported by the low absolute frequencies denoted in the graph. In fact, the most contributing journal is *Plos One* with barely 5.97% (*n* = 4 articles). Additionally, the results revealed that journals from the medical sciences are the main sources in this research area with 86.15% of the total knowledge base (*n* = 56 articles).

## 4. Discussion

The COVID-19 pandemic has forced EDs to elucidate new ways of providing care in a scenario plagued by uncertainty, constant pressure, and restrictions. Not responding effectively to this scenario has resulted in a rising risk of serious health complications in affected patients, the spread of nosocomial infections within the ED wards, and a concerning mortality rate which evidence a lack of concerted intervention where the stakeholders can converge in the search for real efficacious and efficient process improvement programs. A representation of these shortcomings can be noted in the collapse of EDs which has accentuated the need for promoting the design and implementation of emergency care networks or “big hospitals” as an alternative strategy that may address the demand peaks caused by the ongoing outbreak and population dynamics [102,103,104]. This situation is mainly reported in emerging countries where some hospitals have been urged to transfer patients to institutions from other regions [105]. Meanwhile, although The United States and European countries have reported a decline in the total ED visits [106,107,108], the volume of infectious-disease related admissions has substantially risen which entails patient safety challenges and the accentuation of the well-known operational problems identified in [6].

In view of the above, it is necessary to deploy a massive volume of contributions tackling the operational disruptions experienced by the EDs in the overarching pandemic. The review presented here has directed its attention towards the identification of process-improvement strategies that can be leveraged by decision makers and researchers to halt the COVID-19 impact on the emergency care system. In this regard, the ample use of MLDA approaches as solutions to upgrade the response of EDs in this complex scenario (*n* = 41 articles; 63.07%) is evident. This is possible considering the significant fast-growing advance in the implementation of software collecting and storing the sociodemographic and clinical records of patients especially in developed countries [109]. Another motivation related to MLDA popularity is the need for confirming the presence of SARS-CoV−2 based on the symptomatology as well as predicting the health outcomes and resource requirements with sufficient anticipation. In parallel, operational research was found to be the second most contributing domain with *n* = 16 articles (24.61%) which is pertinent with the multifaceted nature of the pandemic and the ED operations. Indeed, Silal and Modelling and Simulation Hub [29] pointed out that OR can help decision makers effectively manage infectious diseases while ensuring efficient resource allocation. Albeit often this discipline is widely applied in healthcare [110], its use has been limited in some way considering the difficulty to extract process data during the current outbreak. Unsurprisingly, the implementation of techniques from the QM area is scant which may be explained by the time that QM projects usually take for their development. More QM contributions are then expected in the coming months and years for increasing the related evidence base. In this regard, the application of Lean Six Sigma can be a fruitful path for research due to its ability for upgrading the supply chain resilience during the COVID-19 era as indicated by Hundal et al. [11]. No less important is the poor involvement of PDI-related studies in the knowledge body. In spite of the multiple protocols that have been crafted for the COVID-19 management within the ED wards, only a few studies show data underpinning their continuing application in the wild. This is a major aspect detected in PDI projects where data-based validation is poorly addressed by healthcare managers.

Multi-objective interventions continue to often be pursued by health decision makers to satisfy stakeholders’ expectations while optimizing constrained resources. The pandemic scenario is not the exception. In light of the literature, 12 objectives have been mainly pursued when increasing the ED performance during the ongoing infection. Moreover, 33.84% of the papers (*n* = 22) have targeted two or more objectives simultaneously. Given the financial restrictions and the need for immediate improvement imposed by this biological disaster, the number of multi-aim projects is expected to rise in the near future. Likewise, the outcomes included in this review are largely variable in view of the complex multi-dimensional nature of ED operations and the new sanitary dynamics imposed by the COVID-19 pandemic. In other words, the ED response has become challenging as new and evolving stages of the COVID-19 have emerged. In this context, each phase has posed different and novel process challenges entailing the creation of new outcomes evidencing the effectiveness of the proposed interventions. Much of them are commonly encountered in studies evidencing the improvement of ED processes during the pre-pandemic period [6,15,23,27]. On a different note, our review revealed that the most targeted objectives have so far been: predicting the health outcomes in COVID-19 patients (n = 32 articles; 49.23%), improving resource allocation (n = 16 articles; 24.61%), and improving the patient flow through the EDs (n = 14 articles; 21.53%). The popularity of the first-ranked aim is motivated by the urgent necessity for comprehending the COVID-19 progression which supports decision making regarding in-time treatment and resource allocation considering patient condition [111]. Thereby, medical and administrative staff can be properly alerted and kept apprised of the potential health consequences if suitable interventions are not shaped and implemented. Going forward, it is possible to anticipate acute worsening of patients including the likelihood of multi-organ failure and death which gives clinicians an effective basis for intervention in the wild. On a different tack, the shortages of different ED resources since the sudden onset of the pandemic have forced these units to correctly search for strategies ensuring their availability, especially in demand peaks. Resource allocation has been therefore prioritized as a cornerstone underpinning the ED response against the climbing volume of admissions. This is even more severe in emerging economies where budget restrictions are commonly addressed when implementing different healthcare improvement plans. When on the verge of the collapse, ED administrators have been pushed towards establishing urgent supplying plans containing new ventilators, beds, oxygen, and medical staff. This reaction, however, has been related to delayed diagnosis and treatment which is widely deleterious when deeming a rapidly evolving disease. These considerations have been also highlighted in [112,113] [8]. However, it is evident that interactions of EDs with upstream and satellite services are poorly considered in the literature which limits the effectiveness of the proposed approaches. There is also a limited number of works focused on optimizing the oxygen allocation in presence of constrained supply. These aspects are critical to avoid deciding which patients receive a specific resource and which patients do not. In this respect, methodologies integrating operations research methods, lean six sigma, and data analytics can give good support for the implementation of rationing evidence-based policies while maximizing value throughout the patient journey. Concerning the third most popular objective, it is important to point out that proper administration of patient flows within the ED may mean the difference between recovery and death for COVID-19 patients. In this regard, [114] concluded that a coordinated system-wide proposal considering telehealth, triage telephone lines, and virtual care may grapple with this crisis. EDs are also called to mine process data as they continuously gather patient health records so that optimization models can be further applied seeking for minimizing flow failures and delays. Combining methods from the MLDA, OR, and CQI domains can serve as a process improvement framework to enhance ED-wide patient flow. It is furthermore essential to use proper demand forecasting methods based on sociodemographic, clinical, and climate predictors to better estimate the demands in future COVID-19 waves whereas vaccination programs advance towards herd immunity. Likewise, it is suggested to develop initiatives targeting the less popular objectives identified in Section 3.2.2.

Some interesting findings arose when exploring the techniques with major use in the reported related literature. For instance, logistic regression and its variations were proved to be the most popular approach when predicting the health outcomes of COVID-19 positive patients (*n* = 26 articles; 81.3%). The main advantage of this technique is the possibility of relating a key probabilistic variable with its predictors so that decision makers can establish the health outcome of a patient if certain variables are fully known with anticipation. Besides, it is feasible to comprehend the effects of each predictor on the response so that targeted interventions can be shaped to combat the COVID-19 course on any patient. The popularity of this method was also noted in the next two most prioritized objectives but to a lesser degree given the mathematical nature of resource allocation and patient flow problems. In these aims, a wide range of approaches has been implemented for mitigating the COVID-19 impact on ED operations. There are, however, some remaining challenging tasks to be tackled in this research field. On the one hand, it is expected to increase the number of studies using hybrid methods for upgrading ED performance. Such integration will provide more valuable insights especially those facilitating the administration of interactions with labs, imaging departments, and upstream healthcare services [115]. In spite of the well-known popularity of simulation techniques in healthcare, its use is still scant in the current COVID-19 crisis. Discrete-event simulation (DES) can be then explored to model healthcare macro-levels, appraise interrelations among services, and pre-test improvement scenarios which would be highly appreciated by local authorities and ED administrators when analyzing future interventions [116] DES models and optimization techniques can also serve as a decision-making platform for optimizing scarce resource allocation, an area of major relevance for ED operational management.

At present, the evolution in the number of contributions through the COVID-19 period shows an increasing trend especially from August 2020 to April 2021. Such behavior has been propelled by the lessons learned from the first waves as well as the dramatic rise in infection and mortality rates, which in some EDs, have reached alarming levels. More studies addressing the 12 objectives identified in this review are needed to create a significant body of knowledge transferable to the EDs which, in light of the results, is at the earlier stages. Despite the substantial efforts that have been made to increase the performance of EDs, it is still necessary to advance the state-of-the-art solutions to be deployed within the emergency care environment. This of course needs to be complemented by external measures halting the COVID-19 progress while in-time and efficient emergency care networks are implemented in the real scenario. Of course, the interventions here exhibited may be adapted and transferred to support the ED operational response in the post-pandemic period. For instance, MLDA techniques used to predict the health outcomes in COVID-19 patients may be employed to foresee the potential health consequences in people suffering from high-prevalent diseases such as cancer and those from the cardiac domain. Meanwhile, the actions plotted to improve resource allocation and patient flow can be adopted by EDs when coping with demand peaks as those forecasted in view of the population growth and new epidemics/pandemics. On a different track, the approaches here proposed may be extrapolated to other healthcare settings such as hospitalization, surgery, and intensive care; for example, MLDA techniques may be implemented to predict the mortality rate, LOS, and the likelihood of home discharge. OR methods could be also used to model patient pathways and treatment options within these services so that inherent resources can be better administered. Likewise, QM and PDI projects may be further delineated to ensure high adherence to healthcare and safety protocols while laying the basis for properly collecting and analyzing data representing the performance of these units.

We acknowledge several limitations in this review. First, the process improvement approaches outlined in this work are restricted to the industrial engineering domain; in this regard, it would be useful to consider methodologies out of this area such as clinical management unit (CMU) (Artiga-Sainz et al. [117], 2021), ABCDE of emergency care, and clinical-related interventions. Secondly, this review did not take financial outcomes into consideration which may fairly limit the application of the approaches described here in EDs where budget is greatly constrained as those located in low-and-middle-income countries. Third, despite using a review process that was carefully implemented and monitored, we cannot discard the possibility that contributing studies may have been excluded. Finally, important insights may have been omitted from the current evidence base in no consideration of the grey literature search.

## 5. Concluding Remarks and Future Directions

The COVID-19 pandemic has challenged the preparedness of emergency departments to deal with disasters in addition to overcoming the operational inefficiencies, predicting the health outcomes in positive COVID-19 patients, improving resource allocation, and expediting the patient flows through EDs. Although extreme and even unpredictable situations can lead emergency departments to disorder, saturation, and even blockage, it is true that similar pandemic situations to the current ones can be repeated in the future. It is therefore necessary to analyze all the approaches used to manage the emergency departments, which will give us a vision of the type of interventions used to set the bases that help to improve them, change them, or seek new actions. The purpose is to increase the response and efficiency of emergency departments under different and exceptional situations.

This article provided a thorough review of the literature using the PRISMA procedure. The resulting evidence base is integrated by 65 articles published in 51 journals from December 2019 to April 2021. The following classification criteria were deemed: (i) contributing research domain, (ii) primary aim, (iii) publication period, and iv) contributing journal. From a holistic perspective, the results revealed a growing evolution of the research field over time with specific publication peaks in December 2020 (10 papers) and March 2021 (8 papers). Despite these efforts, it is noticeable that this area is at the earlier stages and more contributions are expected to emerge in response to the pending gaps.

On a different note, four main knowledge domains were considered due to the multi-causal nature of EDs: operational research (OR), quality management (QM), machine learning and data analytics (MLDA), and protocol design and implementation (PDI). The high involvement of MLDA techniques was noticeable with a participation rate of 61.53%, followed by OR with 24.61%, QM with 9.3%, and PDI with 7.69%. In summary, the logistic regression technique (including multivariable binary regression and logistic regression with post hoc uniform contraction) was proven to be the most contributing technique with a particular focus on predicting the health outcomes in COVID-19 patients. The selected works were also related to 12 different aims targeted when upgrading the ED response during the ongoing pandemic. It was clearly seen that predicting the health outcomes in COVID-19 patients, improving resource allocation, and improving the patient flow through the EDs were found to be the most pursued objectives.

It would be also desirable to work on the following research lines in the future: (i) contributions that integrate logistic regression or its variations with other MLDA, OR, and PDI techniques; (ii) multi-objective considering resource restrictions; (iii) design of strategies for improving patient flow in EDs; (iv) generation of scenarios under uncertainty using metaheuristic techniques, multi-objective programming, and queuing theory; (v) more projects are required for solving the left-without-being-seen problem during the pandemic; (vi) healthcare decision makers are strongly recommended to integrate artificial intelligence techniques with approaches from the operations research (OR) and quality management domains to upgrade the ED performance under socioeconomic restrictions; (vii) more interventions are needed for coping with the less popular objectives identified in Section 3.2; (viii) more works focused on optimizing the oxygen allocation in the presence of limited supply; (ix) it is expected to ramp up the number of studies using hybridization for improving the ED performance against the ongoing COVID-19 and future similar scenarios (x) design and implement simulation approaches for supporting efficacious operations management within EDs during a pandemic; and (xi) review interventions and process improvement approaches that are out of the industrial engineering domain and which may complement those proposed within this paper (i.e., clinical management unit (CMU) (Artiga-Sainz et al., 2021), ABCDE of emergency care, and clinical-related interventions). The techniques cited in these prospect research lines have been widely used in other contexts with very good results and could provide new approaches. It is likely that in the coming months, new papers will appear to delve into these techniques and provide more tools to decision makers, policymakers, ED administrators, and practitioners.

Finally, it must be considered that the problem of EDs can be varied, depending on the limitation of resources (health staff, equipment, infrastructure, etc.), the possibility of expanding resources and the versatility of these, the type of policies for prioritizing/classifying patients, and others. Thus, a certain solution using a specific approach can be very useful in one scenario and little or not at all useful in another. This circumstance forces us to have a wide range of approaches that cover most of the possible situations, seeking concrete solutions to very complex problems.

## Figures and Tables

**Table 1:** Characteristics of the manuscripts selected for the review.

First Author; Year; Country	Sample	Primary Measure of Outcome	Comparator	Key Findings/Conclusion	Quality of the Evidence
Abadi [39] *; 2021; Iran	250	Total deviation from the nurse scheduling constraints	Grasshopper Optimization Algorithm (GOA); Gray Wolf Optimization algorithm (GWO); Cuckoo Optimization Algorithm (COA); Whale Optimization Algorithm (WOA)	HSSAGA outperformed GOA, GWO, COA, and WOA with a total deviation of 561,020. The absolute difference with the above approaches are: 297,722 (GWO), 385,491 (GOA), 388,944 (WOA), and 164,844 (COA).	High
AbdelAziz [40] *; 2020; Saudi Arabia	254	WT; secondary outcome: accuracy	Lexicographic method	The WT for admission passed from 0.0016217 s to 2.48 × 10^−4^ s when using Pareto optimization. Likewise, the accuracy increased from 89% to 97% approximately.	High
Aggarwal [41] **; 2020; India	8 states	Precision; F-Score; Receiver Operating Characteristic (ROC); Precision–Recall (PRC); Matthews Correlation Coefficient (MCC)	TreesJ48; logistics; decision table; ZeroR	The precision, recall, and F-score achieved via the MCDM approach were found to be 0.66, 1.0, and 0.795, respectively. On the other hand, the ROC, PRC, and MCC values were calculated to be 0.5, 0.6, and 0	Moderate
Albahri [42] **; 2021	56	Patient priority	Other MCDM approaches	The maximum patient priority derived from Entropy-TOPSIS was 0.80139 (critical condition) while the minimum was 0.11366 (well health condition)	Moderate
Alfaro-Martinez [43] **; 2021;	1470	Area under curve (AUC)	No intervention	The AUC was found to be 0.8625 and 0.848 for the numerical and categorical scores of the generating cohort, respectively, whereas in the validation cohort, the AUC were 0.8505 and 0.8313 for the same scores.	Moderate
Angeli [44] **; 2021; Italy	301	Area under curve (AUC) for prognosis	No intervention	Integration of clinical and laboratory data increases the CT prognostic value (AUC = 0.841).	Low
Araz [45] ***; 2020; United States	Not specified	Average time for sample collection; availability of testing kits	No intervention	Drive-through COVID-19 testing sites are a strategy to rapidly gather samples from suspected cases with minimal physician-patient contact.	Very low
Assaf [46] **; 2020; Israel	6695	AUC; sensitivity, Positive Predict Value (PPV); Negative Predict Value (NPP); accuracy; F-Score. All these measures are related to risk for critical disease	APACHE II risk prediction score	Having a sensitivity of 88%, specificity of 92.7%, and accuracy of 92% for the critical state of COVID-19 patients, it is demonstrated that the ML models outperformed the APACHE II risk score	Moderate
Balbi [47] **; 2020; Italy	340	Median time from symptom onset to ED admission; prevalence of SARS-CoV−2 infection	No intervention	92% of patients presented in ED obtained a positive RT-PCR while the median time from symptom onset to ED admission was 7 days.	Low
Balmaks [48] **; 2020; Latvia	67	Percentage of failure modes in medium or high risk	No intervention	84.4% of failure modes represent medium or high average risk, with 40.7% being related to organizational factors, 40.7% to individual factors, and 18.5% to environmental factors	Low
Brendish [49] **; 2020; Germany	499	Median time to COVID-19 results	No intervention	Time to results was significantly lower in the testing group than in the control group (hazard ratio 4023 (95% CI 545–29 696), *p* < 0.0001).	Low
Bolourani [50] *; 2021; United States	11,525	Mean accuracy; Area under curve (AUC)	Modified Early Warning Score, XGBoost + SMOTEENN, logistic regression	The XGBoost method evidenced the highest mean accuracy (0.919) while the AUC was found to be 0.77 (standard deviation = 0.05).	High
Carlile [51] ***; 2020; United States	1855	AUC; accuracy; sensitivity; specificity; percentage of healthcare workers agreed on the easiness of AI implementation in their workflow	No intervention	The resulting AUC was 0.854 while the accuracy, sensitivity, and specificity were 81.6%, 82.8%, and 72.6%, respectively. Likewise, 86% of the healthcare workers agreed on the fact the AI model was easy to implement in their workflow.	Very low
Casiraghi [52] **; 2021; Italy	301	Area under curve (AUC), sensitivity; specificity; F1 score; accuracy	Generalized linear models	The risk prediction results evidenced a reduction in accuracy by an average of 0.06 concerning the five performance measures (AUC from 0.81 to 0.76, sensitivity from 0.72 to 0.66, specificity from 0.76 to 0.71, F1 score from 0.62 to 0.55, accuracy from 0.74 to 0.68).	High
Chen [53] **; 2020; China	2863	Time from pre-examination to virus screening; hospital visiting time; waiting time for consultation Secondary outcomes: median waiting time for image examination; moving distance	No intervention	The time from pre-examination to virus screening was reduced from 34 to 3 h, the visiting time was decreased from 18 to 8 h, and the WT for a consultation was narrowed from 2 h to 10 min. in Addition, the median WT for image examination was slackened from 40 to 3 min. Finally, the moving distance passed from 800 to 10 min.	Low
Chopra [54] **; 2020; United States	323	Median time to revisit; median hospital LOS	No intervention	A total of 8 were discharged from the ED during their index visit and 225 were admitted to the hospital. Among those discharged, 25/98 (25.5%) returned within 28 days of index ED presentation	Very low
Chou [55] **; 2021; United States	580	AUC; AP; accuracy; F1-Score; kappa; recall (sensitivity); specificity; PPV (precision); NPV; ROC. All these measures for confirmed diagnosis of COVID-19	No intervention	The three methods, Random Forest outperformed the others with an AUC of 0.86, followed by Gradient Boosting with 0.83, and Extra Trees with 0.82.	Low
Diep [56] **; 2021; Belgium	745	Area under curve (AUC)	No intervention	The AUC for the predictive model was calculated to be 0.931 (95% CI: 0.910–0.953) with a standard error of 0.010	Low
De Moraes [57] *; 2020; Brazil,	235	Area under curve (AUC); sensitivity; specificity; Brier score	No intervention	Support Vector Machine was found to produce the best performance (AUC: 0.85; sensitivity: 0.68; specificity: 0.85; Brier score: 0.16)	Moderate
De Nardo [58] **; 2020; Italy	10	Patient priority	No intervention	The maximum observed score (critical condition) was 69% while the minimum (well health condition) was 15%	Low
Esposito [59] *; 2021; Italy	77	Area under curve (AUC)	No intervention	Moderate AUC of 0.76, 0.75, and 0.77 for well-aerated lung, semi-consolidation, and consolidation predicted worst hypoxemia during hospitalization correspondingly.	Moderate
Feng [60] **; 2021; China	132	AUC; F1-Score; specificity; recall; precision (for early identification of COVID-19 in ED admission)	No intervention	The LASSO model performance in the testing set and the validation cohort resulted in AUC (0.841 and 0.938), the F−1 score (0.571 and 0.667), the recall (1.000 and 1.000), the specificity (0.727 and 0.778), and the precision (0.400 and 0.500)	Moderate
Freund [61] **; 2020; Italy, Spain, France, Chile, Belgium, and Quebec.	3358	AUC; sensitivity	No intervention	Whole population: AUC = 0.79, 95% CI = 0.76 to 0.81. COVID-19 patients: AUC = 0.81, 95% CI = 0.77 to 0.85.	Low
Garbey [62] **; 2020; French	50 per day	Death rate due to COVID-19	No intervention	After calibrating the Markov model, the death rate was found to be 25% approximately.	Moderate
García de Guadiana-Romualdo [63] **; 2021; Spain	99	AUC; accuracy; sensitivity; specificity (For predicting 28-day mortality)	No intervention	MR-proDAM showed the highest AUC for predicting mortality and progression to severe disease. 25.3% of the cases developed into serious diseases, and the 28-day mortality rate was 14.1%.	Low
Gavelli [64] **; 2021; Italy	480	Death adjusted hazard ratio	Multivariable logistic regression; Cox regression hazard model	When in-hospital mortality was assessed, a meaningful gap was evident between scores of 0–1 and 2 vs. 3 and 4–5. Specifically, the death adjusted Hazard Ratio for Novara-COVID scores of 3 and 4–5 were 2.6 (1.4–4.8) and 8.4 (4.7–15.2), correspondingly.	Moderate
Goodacre [65] **; 2021; United Kingdom	11,773	AUC; ROC; C-Statistic; sensitivity; specificity	NEWS2 Score	C-statistic of 0.80 (95% confidence interval 0.79–0.81), sensitivity 0.98 (0.97–0.98), and specificity 0.34 (0.34–0.35)	Moderate
Gordon [66] **; 2020; United States	295	Percentage of patients triggering a symptom alert	No intervention	Of the 210 who completed at least one questionnaire, only 72/210 (34%) triggered a symptom alert to the central nursing pool during their monitoring enrollment period, and only 15% (315/2161) of questionnaires across all patients triggered an alert to the central nursing pool	Moderate
Haddad [67] ***; 2021; England	29 hospitals	Mean number of shortages	No intervention	The optimization model led to a 55% reduction in the number of shortages	Moderate
Heldt [68] *; 2021; England	1235	AUC; sensitivity; specificity; Brier score; precision-recall	No intervention	Logistic regression reaches an AUC of 0.70, the random forest 0.77 and XGBoost reach 0.76. However, all models showed improved accuracy with F1 scores of 0.56–0.61	High
Joshi [69] **; 2020; United States	390	C-statistic; sensitivity	No intervention	The C-statistic was found to be 78% with an optimized sensitivity of 93%. By constraining PCR testing to predict COVID-19 patients, it would be possible to achieve a 33% increase in the allocation of testing resources.	Moderate
Kim [70] **; 2020; Korea	184	Percentage of COVID-19 patients with fever before OHCA; percentage of COVID-19 patients with pneumonic infiltration	No intervention	55.6% of patients in the COVID-19-positive group had a fever before out-of-hospital cardiac arrest (OHCA) and 16.9% of the COVID-19-negative group had a fever before OHCA (*p* = 0.018). A total of 88.9% patients in the COVID-19-positive group had a chest X-ray indicating pneumonic infiltration.	Low
Kirby [71] *; 2021; United States	90,549	C-statistics for in-hospital all-cause mortality; hospital admissions	Charlson Comorbidity Index (CCI) and Elixhauser Comorbidity Index (ECI)	C-statistics of COVID-related high risk chronic condition predicting in-hospital all-cause mortality was 0.73 (0.69–0.76)	Moderate
Kline [72] *; 2021; United States	19,850	Prevalence of SARS-CoV−2 infection; area under curve (AUC)	No intervention	In the validation sample (*n* = 9975), the probability from logistic regression score produced an area under the receiver operating characteristic curve of 0.80 (CI: 0.79–0.81). On the other hand, the pooled prevalence of infection among those tested was 34%.	Moderate
Lancet [73] **; 2021, United States	1673	LOS; in-hospital mortality; likelihood of survival to discharge	No intervention	The median hospital LOS was 6 days (IQR: 2–11 days) while 34.5% of the patients died. In younger patients, the likelihood of survival to discharge was 1.68 (95% CI, 1.49–1.88; *p* <0.001).	Low
Levine [74] *; 2021; United States	1014	C-statistic; sensitivity; specificity (for predicting a 14-day period	No intervention	It obtained a sensitivity of 83% and specificity of 82%, counting with C-statistics for derivation 0.8939 (95% CI, 0.8687 to 0.9192) and validation 0.8685 (95% CI, 0.8095 to 0.9275).	High
Liu [75] **; 2020; China	643	AUC; sensitivity; specificity; accuracy; median survey time	Conventional ED track	AUC of 0.99, sensitivity of 94.1%, specificity of 95.1%, and accuracy of 94.6% using the training data set. The median survey time without the model in the quarantine station was 100.5 min (95% CI 40.3–152.5), vs. 34 min with the model in the quarantine station (95% CI 24–53; *p* <.001).	Moderate
McDonald [76] **; 2020; United States	1026	Prevalence of SARS-CoV−2 infection; area under curve (AUC)	No intervention	The COVID-19 prevalence was 9.6% whereas AUC of 0.89 (95% CI = 0.84–0.94)	Moderate
Mehrotra [77] **; 2020: United States	Not specified	Number of ventilators in stock	No intervention	If more than 40% of the existing ventilator inventory is available for COVID-19 patients, the national stockpile is approximately enough to satisfy the demand in mild cases. Nevertheless, if less than 25% of the current ventilator inventory is available for COVID-19 patients, the national stockpile and the projected production could not address the peak demands caused by the pandemic.	Moderate
Mitchell [78] ***; 2020; Papua New Guinea	210 per day	Satisfaction level on the new triage and flow system as a way of identifying the most urgent patients	No intervention	A total of 100% of the respondents agreed on the fact that the new triage and flow system has helped in the identification and prioritization of new patients.	Very low
Möckel [79] **; 2021; Germany	1255	Area under curve (AUC) representing the need for mechanical ventilation during index stay or after readmission; median LOS	No intervention	A sufficient discriminatory power (C-index 0.75) was achieved for predicting the need for artificial ventilation or death within 14-day period after ED admission	Moderate
Moss [80] **; 2020; Australia	Not specified	Mean bed time	No intervention	The mean bed time was found to be 8 days	Low
Nepomuceno [81] ***; 2020; Brazil	Not specified	Number of beds feasible to be evacuated and reallocated to COVID-19 patients	No intervention	In summary, 3772 beds are feasible to be evacuated and reassigned for new COVID-19 cases in one year considering different interventions on surgery and patient LOS.	Low
Nguyen [82] *; 2020; France	334	C-statistics (need for artificial ventilation or death within 14-day period after ED admission)	No intervention	A sufficient discriminatory power (C-index 0.75) was achieved for predicting the need for artificial ventilation or death within 14-day period after ED admission	High
O’Reilly [83] ***; 2020; Australia	Not specified	Number of ventilator-free days, hospital length of stay and death during hospital admission.	No intervention	The COVED protocol for addressing the operational consequences of the COVID-19 pandemic	Very low
Parker [84] *; 2020; United States	75 hospitals	Surge capacity	No intervention	An 85% reduction in required surge capacity was achieved considering uncertainties inherent to the COVID-19 pandemic.	High
Peng [85] **; 2020; Canada	39,525	WT; LOS	No intervention	After simulating the proposed alternatives, the maximum reduction percentage in WT and LOS were 76.33% and 31.16%.	High
Plante [86] *; 2020; United States	192,779	Area under curve (AUC)	No intervention	AUC was found to be 0.91 (95% CI 0.90–0.92).	Moderate
Retzlaff [35] ***; 2020; United States	Not specified	Number of COVID-19 tests processed per day	No intervention	The laboratory was calculated to process 30 tests per day.	Very low
Romero-Gameros [87] ***; 2021; Mexico	2173	Prevalence of SARS-CoV−2 infection; sensitivity; specificity	No intervention	A prevalence of 53.72% of SARS-CoV−2 infection was detected. The symptom with the highest sensitivity was cough 71%, and a specificity of 52.68%	Low
Saegerman [88] **; 2021; Belgium	2152	Area under curve (AUC)	No intervention	The resulting area under curve was 0.71 (95% CI: 0.68–0.73)	Low
Sangal [36] ***; 2020; United States	190,000	Number of provider shifts; contact time between physician and COVID-19 patients	No intervention	The provider shifts decreased by 42% whereas the contact time between physician and COVID-19 patient was reduced by 66%	Low
Shamout [89] *; 2020; United States	19,957	AUC; sensitivity; specificity; PPV; NPV; F1-score (for predicting deterioration within 96 h)	Imaging reading via two experienced chest radiologists	AUC of 0.786 (95% CI: 0.745–0.830) for prediction of deterioration within 96 h	High
Sherren [90] ***; 2020; United Kingdom	316	Percentage of patients survived to critical care discharge	No intervention	Of the 201 patients received in the ED with a completed critical care status, 71.1% survived to critical care discharge.	Low
Suh [91] ***; 2020; United States	1832	Number of patients discharged with oxygen concentrators for use at home (period: 2 months); number of patients discharged with pulse oximeters	No intervention	In this case, 1040 patients were discharged with pulse oximeters and 792 patients were discharged at home with portable oxygen concentrators.	Low
Sung [92] **; 2020; United States	656	AUC; sensitivity; specificity; PPV; NPV	No intervention	Risk score of ≥3 in the development cohort (sensitivity = 85.1%; specificity of 75.0%; PPV = 71.8% and NPV = 87.0%); in the validation cohort (sensitivity = 79.6%; specificity = 70.9%). AUC = 0.87 (95% confidence interval (CI) 0.82–0.92) in the development cohort and 0.85 (95% CI 0.78–0.92) in the validation cohort.	Moderate
Tang [93] **; 2020; United States	28,454 standard patients and 1693 COVID-19-like illness	Left-without-being-seen rates (LWBSR); LOS	No intervention	After adopting a one-floating provider configuration, the average LOS was reduced by 24.34% for discharged patients and 13.91% for hospitalized patients while LWBSR was slackened by 84.57%	High
Teklewold [94] ***; 2020; Ethiopia	Not specified	Number of failure modes associated with no transmission-based precautions	No intervention	A total of 12 out of 22 failure modes were found to be related to non-adherence to transmission-based precautions.	Low
Van Klaveren [95] *; 2020; Netherlands	5912	AUC; sensitivity; specificity; accuracy; PPV; NPV (for the COVID-19 outcome prediction in the ED)	No intervention	AUC in 4 hospitals: 0.82 (0.78; 0.86); 0.82 (0.74; 0.90); 0.79 (0.70; 0.88); 0.83 (0.79; 0.86)	Moderate
Van Singer [96] **; 2021; Switzerland	76	30-day intubation/mortality, and oxygen requirement via AUC	No intervention	The highest accuracy for 30-day oxygen requirement (AUC 0.84; 95% CI, 0.74–0.94).	Low
Wang [97] **; 2021; United States	542	Percentage and AUC of COVID-19 patients with need for transfer to ICU within 24 h of ED admission	No intervention	A total of 10% of COVID-19 patients required transfer to ICU within 24 h of ED admission. On the other hand, the AUC was found to be 0.54 (standard error 0.02, CI 0.50–0.59)	Low
Zeinalnezhad [98] **; 2020; Iran	Not specified	WT	No intervention	The second simulated scenario (hiring more reception staff while assigning free human resources in other wards) led to a 62.3% reduction in patient waiting time.	Moderate
Zhang [99] **; 2021; China	500	Median time; average LOS; transfer density	No intervention	The median time for each state were the following: state 1 (pre-infection period): 0.26 days, state 2 (acute infection period): 6.13 days, state 3 (pre new coronary pneumonia): 1.05 days. On the other hand, the average LOS for each state were as follows: state 1: 2.14 days, state 2: 5.22 days, and state 3: 6.64 days. Finally, the transfer densities were: state 1: 5.54, state 2: 0.13, and state 3: 0.57.	Moderate
Zhang & Cheng [100] ***; 2020; United States	42,309	Weekly infection rate in healthcare workers and patients	No intervention	The weekly infection rate in healthcare workers and patients was reduced from 3–5.9%, to 1–2.1%.	Moderate
Zhou [101] **; 2021; China	174	Time from illness onset to hospital admission	No intervention	In non-survivors, the time from illness onset to hospital admission was 10.0 (7.0–14.0) days whereas in survivors was 10.0 (7.0–13.0) days	Low

Low risk (*), Unclear risk (**), High risk (***).

**Table 2:** Papers demonstrating the use of operational research techniques for augmenting the emergency department response to the COVID-19 pandemic.

Authors	Technique Type
Single	
Tang et al. [93]	Discrete event simulation
Nepomuceno et al. [81]	Data envelopment analysis (DEA)
Mehrotra et al. [77]	Stochastic optimization
AbdelAziz et al. [40]	Multi-objective pareto optimization
Peng et al. [85]; Moss et al. [80]	Simulation
Aggarwal et al. [41]	Additive utility assumption
Araz et al. [45]	System dynamics
Hybrid	
Garbey et al. [62]	Markov chains, stochastic optimization
Albahri et al. [42]	Entropy, TOPSIS
De Nardo et al. [58]	Potentially all pairwise ranking of all possible alternatives (PAPRIKA), multi-criteria decision making (MCDM)
Parker et al. [84]	Linear programming, mixed-integer programming
Zeinalnezhad et al. [98]	Colored petri nets, discrete event simulation
Zhang & Cheng. [100]	Logistic regression, Markov chains
Abadi et al. [39]	Hybrid salp swarm algorithm and genetic algorithm (HSSAGA)
Haddad et al. [67]	Simulation, optimization

**Table 3:** Papers demonstrating the use of quality management techniques for augmenting the emergency department response to the COVID-19 pandemic.

Authors	Technique Type
Single	
Chen et al. [53]	Lean Manufacturing
Casiraghi et al. [52]; Teklewold et al. [94]; Balmaks et al. [48]	FMEA
Hybrid	
Retzlaff [35]	Critical pathways, lean manufacturing
O’Reilly et al. [83]	Logistic regression, survival regression, linear regression, continuous quality improvement

**Table 4:** Papers demonstrating the use of machine learning and data analytics techniques for augmenting the emergency department response to the COVID-19 pandemic.

Authors	Technique Type
Single	
Chopra et al. [54]; Sung et al. [92]; Alfaro-Martinez et al. [43]; Kirby et al. [71]; Lancet et al. [73]	Multivariate logistic regression
Nguyen et al. [82]	Multivariate cox proportional hazard model
Joshi et al. [69]; Kim et al. [70]; Levine et al. [74]; Wang et al. [97]; Angeli et al. [44]; Zhou et al. [101]	Logistic regression
Liu et al. [75]	Artificial intelligence
Freund et al. [61]	Multivariate binary logistic regression
Brendish et al. [49]; Esposito et al. [59]	Cox proportional hazards regression
Gordon et al. [66]	Mixed-effect logistic regression
García de Guadiana-Romualdo et al. [63]	Multivariate regression
Kline et al. [72]	Stepwise forward logistic regression
Carlile et al. [51]	Deep learning
Plante et al. [86]	Gradient boosting
Hybrid	
Shamout et al. [89]	Deep neural network, gradient boosting
Balbi et al. [47]	Poisson regression, logistic regression
Van Klaveren et al. [95]	Logistic regression with post hoc uniform shrinkage
De Moraes et al. [57]	Neural networks, random forest, gradient boosting, logistic regression, support vector machine (SVM)
McDonald et al. [76]; Heldt et al. [68]	Logistic regression, random forest, and gradient-boosted decision tree
Zhang & Cheng [100]; Zhang et al. [99]	Logistic regression, Markov
O’Reilly et al. [83]	Logistic regression, survival regression, Linear regression, continuous quality improvement
Assaf et al. [46]; Chou et al. [55]	Neural network, random forest, classification and regression decision tree (CRT)
Van Singer et al. [96]; Möckel et al. [79]	Logistic regression and CRT
Diep et al. [56]	Logistic regression, Mann–Whitey, chi-cuadrado
Saegerman et al. [88]	Binary logistic regression and bootstrapped quantile regression, classification and regression tree analysis.
Romero-Gameros et al. [87]	Logistic regression, Mantel–Haenszel
Bolourani et al. [50]	Artificial intelligence, logistic regression, XGBoost combines a recursive gradient-boosting method called Newton boosting, with a decision-tree model, decision making
Goodacre et al. [65]; Feng et al. [46]	Multivariable regression with least absolute shrinkage and selection operator (LASSO)
Gavelli et al. [64]	logistic regression and cox regression

**Table 5:** Papers demonstrating the design and implementation of healthcare protocols for augmenting the emergency department response to the COVID-19 pandemic.

Authors	Technique Type
Single	
Suh et al. [91]; Sangal et al. [36]; Sherren et al. [90]	Critical pathways
Mitchell et al. [78]	Intelligent integrated triage tool
Hybrid	
Retzlaff [35]	Critical pathways, lean manufacturing

## Data Availability

In this case, no new data were created or analyzed in this study. Therefore, data sharing is not applicable to this article.
